# Pure Red Cell Aplasia (PRCA) and Cerebellar Hypoplasia as Atypical Features of Polyglandular Autoimmune Syndrome Type I (APS-1): Two Sisters With the Same AIRE Mutation but Different Phenotypes

**DOI:** 10.3389/fped.2019.00051

**Published:** 2019-02-26

**Authors:** Matteo Chinello, Margherita Mauro, Gaetano Cantalupo, Rita Balter, Massimiliano De Bortoli, Virginia Vitale, Ada Zaccaron, Elisa Bonetti, Rossella Gaudino, Elena Fiorini, Simone Cesaro

**Affiliations:** ^1^Pediatric Hematology Oncology, Azienda Ospedaliera Universitaria Integrata, Verona, Italy; ^2^Mother and Child Department, University of Verona, Verona, Italy; ^3^Child Neuropsychiatry, University of Verona, Verona, Italy; ^4^Pediatric Division, Department of Pediatrics, University Hospital of Verona, Verona, Italy

**Keywords:** polyglandular autoimmune syndrome type I, pure red cell aplasia (PRCA), hematopoietic stem cell transplantation (HCT), cerebellar hypoplasia, acute disseminated encephalomyelitis (ADEM)

## Abstract

The polyglandular autoimmune syndrome type I is a rare hereditary autosomal recessive disease. We describe a child with the classic triad of the disease and her sister with pure red cell aplasia and cerebellar hypoplasia. The latter received two haematopoietic stem cell transplantations, complicated by an acute disseminated encephalomyelitis.

## Introduction

The polyglandular autoimmune syndrome type I (APS-1), also known as autoimmune polyendocrinopathy-candidiasis-ectodermal dystrophy (APECED), is a rare hereditary autosomal recessive disease characterized by the classic triad of adrenal insufficiency (Addison's disease), hypoparathyroidism, and chronic mucocutaneous candidiasis. The disease may be associated with other autoimmune disorders such as hypogonadism, type I diabetes mellitus, panhypopituitarism, pernicious anemia, chronic active hepatitis, pancreatitis, pneumonitis, nephritis, malabsorption syndrome, vitiligo, alopecia, enamel dysplasia, nail dystrophy, retinitis, keratopathy, and pure red cell aplasia (1, 2). The disease is caused by mutations in the autoimmune regulator gene *AIRE* (3, 4). This gene was mapped at 22q22.3 and consisted of 14 exons (4). More than 100 different mutations have been reported (2). The prevalence is ~1:100,000 with a higher prevalence in some countries, such as Finland (1:25,000) (2). Autoimmune pathogenesis is supported by the detection of organ-specific autoantibodies and autoantibodies to type 1 interferons (interferon-α and interferon-ω) (5, 6). The absence of autoantibodies does not rule out the presence of a specific organ antibody-mediated damage, as autoantibodies may disappear once the target organ has been completely destroyed (7). Association between APS-1 and pure red cell aplasia (PRCA) is rare: only 8 cases have been described in the literature (8–14). We describe two Caucasian sisters with the same *AIRE* mutation, but different phenotypic expression of APS-1. The older sister has the classic triad with adrenal insufficiency, hypoparathyroidism, and chronic mucocutaneous candidiasis while the younger one has a partial adrenal failure associated with PRCA and mild cerebellar hypoplasia; the latter received two haematopoietic stem cell transplantations, both complicated by graft failure. The occurrence of autoimmune demyelinating encephalitis after the second haematopoietic stem cell transplantation (HSCT) is also described.

## Case Report

### Case 1

**Figure 1:**
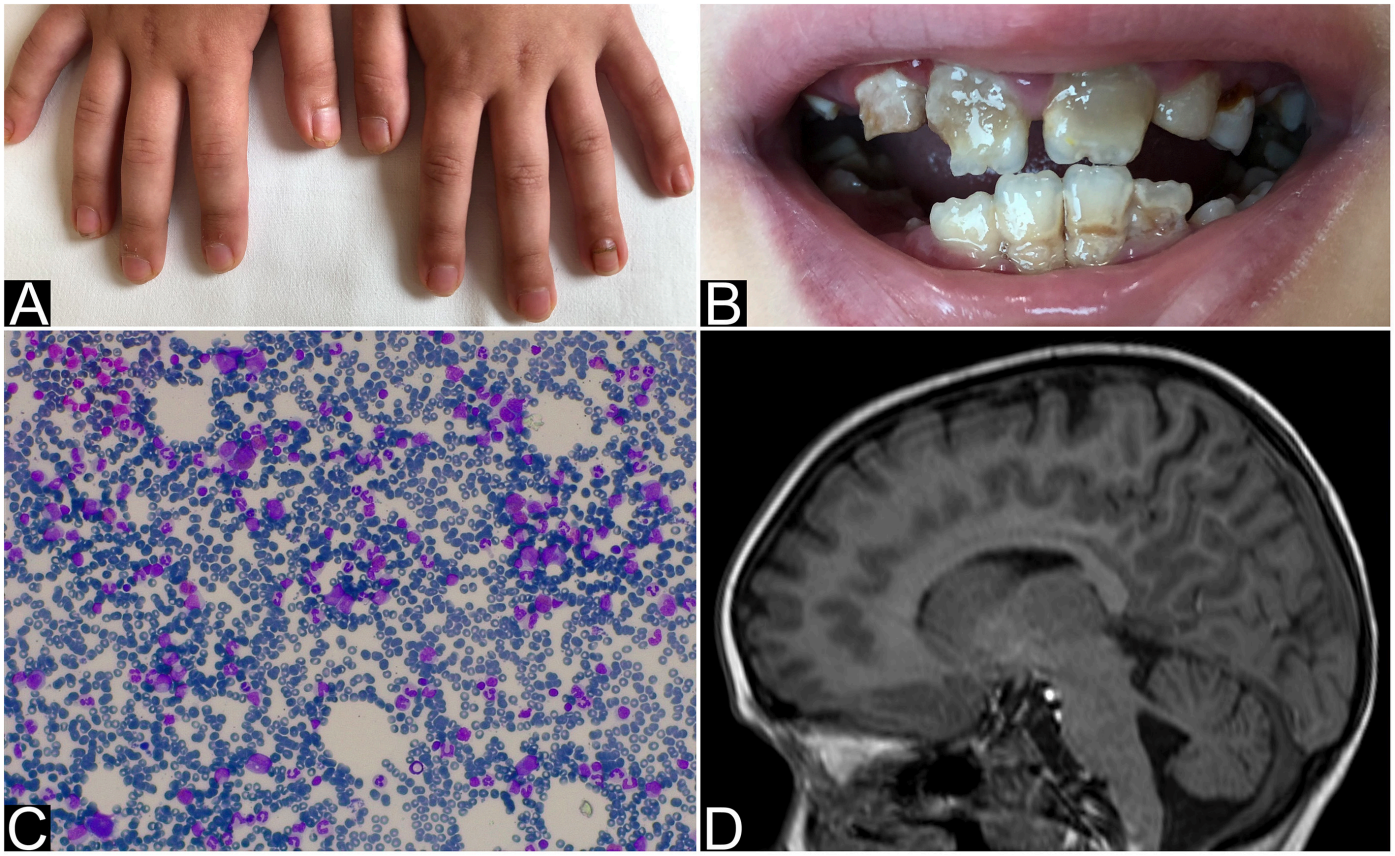
Nails and teeth of patient 1. Bone marrow aspirate and brain MRI of patient 2. **(A)** Nail dystrophy with candidiasis and **(B)** enamel hypoplasia (amelogenesis imperfecta) in patient 1. **(C)** May-Grunwald-Giemsa-stained bone marrow smear (×40 magnification) showing the absence of erytrhoid precursors with normal component of myeloid precursors and lymphocytes and **(D)** MRI that shows mild hemispheric and vermian cerebellar hypoplasia in patient 2.

An 8 year-old Ukrainian female, sister of case 2, was referred to our Emergency Department for fever, vomit, and abdominal pain while she was in Italy together with her parents, who were assisting her sister for allogeneic HSCT. The patient was born after a full-term gestation, from non-consanguineous parents, the birth weight being 3,000 grams. The patient first presented chronic nail candidiasis when she was 2 year old, followed by oral candidiasis at 3. At the age of 5, she developed seizures that were treated with anticonvulsant therapy (levetiracetam and lamotrigine). When she was 6 years old, primary adrenal failure was diagnosed and hydrocortisone replacement therapy was started. Growth retardation was reported from the age of 6. Physical examination when the child came to our attention: weight 16.7 Kg (<3°p), height 115 cm (−2 DS), painful abdomen, and oral-nail candidiasis (Figure 1A). Blood exams showed a slight increase of white blood cells (WBC) and inflammatory indices [WBC 17.320/mm^3^, polymorphonuclear cells (PMN) 14.350/mm^3^, C-reactive protein (CRP) 29 mg/dL] associated with severe hyponatremia and hypocalcemia (Na 112 mmol/L, Ca 1.64 mmol/L). Parathormon (PTH) resulted <0.26 pmol/L (nv 1.00–8.00). Hydrocortisone and fludrocortisone were administered intravenously at first and a slow intravenous correction of electrolytes was started. The association of chronic mucocutaneous candidiasis, adrenal insufficiency and hypoparathyroidism led to the diagnosis of APS-1 which was confirmed by AIRE mutation: homozygous mutation in exon 6: c.769C>T (p.Arg257^*^). An extended diagnostic assessment was performed to rule out any possible associated manifestation. Eye examination revealed bilateral autoimmune keratitis and dental evaluation showed enamel hypoplasia (amelogenesis imperfecta) (Figure 1B). The reported seizures were most likely secondary to hyponatremia and hypocalcemia. For this reason, an electroencephalography (EEG) was performed, showing no abnormalities. Hydrocortisone was adapted to sodium levels. Treatment also included supplementation with calcitriol and calcium for hypoparathyroidism and antimycotic therapy with fluconazole for chronic mucocutaneous candidiasis. Currently the child is in good clinical condition with normal blood tests.

### Case 2

The 6 year-old younger sister was referred to our center from Ukraine in order to perform HSCT for PRCA, diagnosed at the age of 3. The patient was born after a full-term gestation. The birth weight was 3,300 grams. At the age of 3 the patient was hospitalized for severe paleness and an isolated normochromic normocytic anemia was detected [hemoglobin 5 g/dl, mean corpuscular volume (MCV) 85 fL (normal value 75.0–85.0), mean corpuscular hemoglobin (MCH) 29.0 pg (normal value 25.0–35.0), reticulocytes 1.1%, WBC 11.400/mm^3^, PMN 5.800/mm^3^, platelets count 163.000/mm^3^]. The immunocytology study of peripheral blood lymphocyte subpopulations showed a slight increase in the content of T-lymphocytes with a normal CD4 +/CD8 + ratio, and a moderate decrease (as a percentage and as absolute value) of natural killer cells. Physical examination was normal. Bone marrow aspirate and biopsy showed complete absence of erythroid lineage, normal granulocyte and megakaryocyte, absence of blast cells and signs of myelodysplastic syndrome (Figure 1C). Bone marrow cytogenetics (fluorescent *in situ* hybridization): negative for trisomy 8, trisomy 21, monosomy 5 or deletion of long arm of chromosome 5 (−5 q). No monosomy 7 nor deletion of long arm of chromosome 7 (−7 q), and no disorder of the sex chromosomes were identified. An extensive diagnostic work-up excluded other common causes of anemia: Diepoxybutane test for Fanconi anemia, the detection of paroxysmal nocturnal haemoglobinuria (PNH) clone, the Adenosine Deaminase enzyme activity and the study of genetic mutations for Diamond-Blackfan anemia resulted negative or normal. Moreover, an autoimmune lymphoproliferative syndrome was excluded by the search of double negative T lymphocytes that resulted normal: 0.9% (nv < 1.7%). The culture of haematopoietic progenitor cells in Colony Forming Unit (CFU) assays showed a cell growth under the normal range. Serological test did not revealed any evidence of recent parvovirus B19, cytomegalovirus, Human herpesvirus 6, Human immunodeficiency virus, hepatitis B virus, hepatitis C virus, or Epstein-Barr virus infection. Direct and indirect Coombs' tests, anti-nuclear antibody, anti-dsDNA, and Vitamin B12 were normal. The patient received initially therapy with intravenous immunoglobulin, corticosteroid, and erythropoietin without efficacy. The patient was treated once every 7–10 days with red blood cells (RBC) transfusion associated with chelation therapy. Based on the unresponsiveness to the therapy and the transfusion dependency, the patient was candidate for allogeneic HSCT. During pre-transplant work-up the brain Magnetic Resonance Imaging (MRI) showed a mild cerebellar hypoplasia (Figure 1D) and routine blood exams showed severe hyponatremia (sodium 122 mmol/L) that had never been highlighted before. The presence of hyponatremia and the recent diagnosis of APS-1 in her sister led to suspect the same diagnosis in this patient and a search for mutations of the AIRE gene was launched. Indeed she underwent hormonal tests that revealed partial adrenal failure (normal basal cortisol 559 nmol/L (normal value 120–620), high basal ACTH 339.28 pg/mL (normal value 8.11–59.53), normal response to ACTH stimulation test, without hypoparathyroidism nor any other hormonal alteration. Therefore, therapy with hydrocortisone was started. Considering the transfusion dependency, the patient proceeded with the scheduled HSCT from an unrelated bone marrow donor.

**Figure 2:**
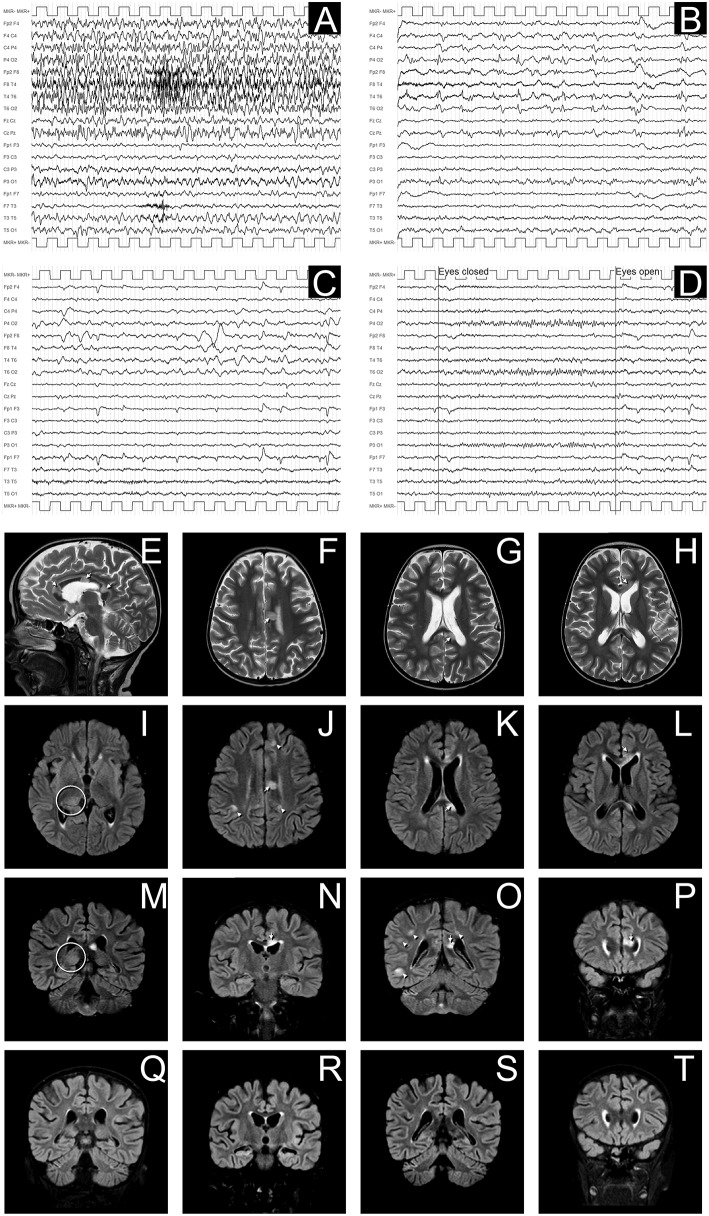
EEG and MRI evolution of encephalitic episode. **(A–D)** EEG recordings in longitudinal bipolar montage, 15 s each panel, calibration (MKR) amplitude 100 μV. In **(A)** a clear-cut ongoing epileptic discharge involving the right hemisphere (mainly the infrasylvian derivations) is documented, clinically associated with the sub-acute onset of neurological symptoms, consistent with the diagnosis of focal Non-Convulsive Status Epilepticus (NCSE). In **(B)** the recording obtained 8 h later, after intravenous levetiracetam and phenytoin treatment, shows the resolution of the NCSE with residual diffuse slowing (predominant on the right hemisphere) and periodic temporo-occipital sharp transients (more evident on P4-O2, T4-T6, and T6-O2 channels). Ten days after sub-acute onset, the EEG does not show epileptiform discharges, but an increased amount of slow activity remains, with obvious preponderance on the right temporal regions **(C)**. In **(D)** the EEG recording performed after 3 months demonstrates a complete recovery, with normal reactivity of the posterior background alpha rhythm. **(E–P)**: Brain MRI images obtained at the onset of neurological symptoms, demonstrating multiple focal hyperintense lesions in T2-weighted (sagittal plane in **E**; axial images in **F–H**) and FLAIR (axial images **I–L**; coronal plane in **M–P**) sequences. The largest 3 lesions were located in corpus callosum (arrows in **E–H**,**J–L**,**N–P**). A clear hyperintensity was also visible on the right posterior thalamus (circles in **I,M**). Multiple lesions were evident at the cortico-subcortical junction bilaterally (arrowheads in **J,O**), mainly involving the right hemisphere, being the largest in the right temporo-occipital region **(O)**. The MRI performed 30 days after the encephalitic episode showed a complete resolution of the focal lesions (in panels **Q–T** are depicted the correspondent coronal FLAIR images as in **M–P**).

**Figure 3:**
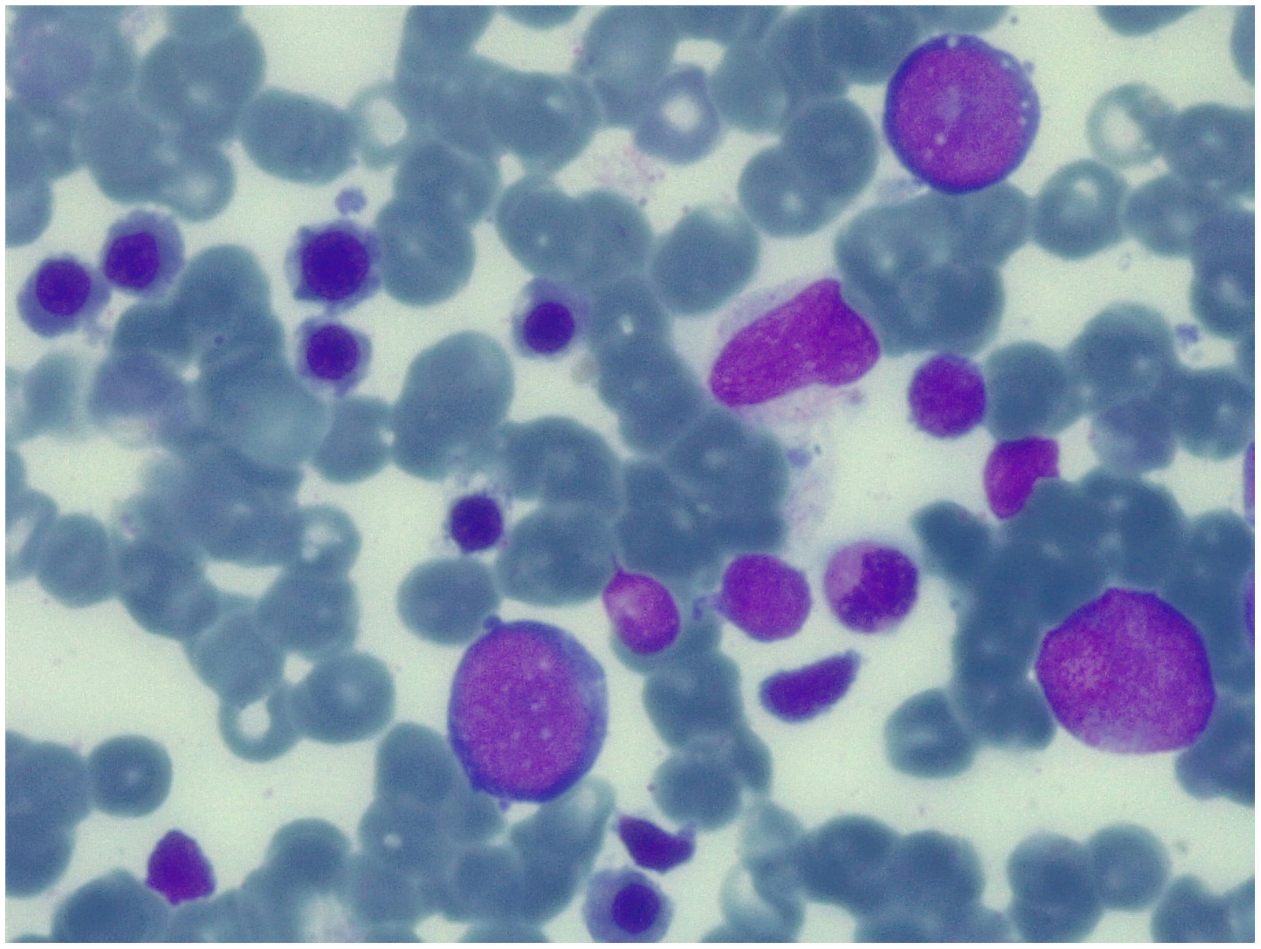
Bone marrow aspirate at 100 days after the second transplant. May-Grunwald-Giemsa-stained bone marrow smear (×100 magnification) showing normal trilineage haematopoiesis with the presence of erythroid precursors.

Conditioning regimen was based on treosulfan (3 × 12 g/m^2^/day), fludarabine (5 × 30 mg/m^2^/day), thiotepa (2 × 5 mg/kg), and rabbit antithymocyte globulin (ATG Neovii^TM^) (3 × 13 mg/kg/day). The composition of the graft was: total nucleated cell (TNC) 5.66 × 10^8^/kg and CD34+ 5.55 × 10^6^/kg. Graft vs. host disease (GVHD) prophylaxis was based on cyclosporine and short term methotrexate. The result of AIRE mutation arrived during the first month of HSCT, confirming that the girl presented the same AIRE mutation as her sister (homozygous mutation in exon 6: c.769C>T (p.Arg257^*^). Meanwhile, after a transient increase of neutrophil count on day + 19 (peak neutrophil count of 6.920/mm^3^), the patient presented a progressive decrease of neutrophil count and the chimerism analysis performed on day + 26 confirmed the graft failure (0% donor-derived cells). Given the patient good clinical conditions, a second transplant from the same unrelated donor was scheduled 42 days after the first transplant. Considering the complete bone marrow aplasia, a reduced intensity conditioning regimen, similar to that recommended for severe aplastic anemia, was used: rabbit antithymocyte globulin (ATG Genzyme^TM^) (3 × 3.3 mg/kg/day), cyclophosphamide (4 × 30 mg/kg/day), fludarabine (4 × 30 mg/ m^2^/day), while GVHD prophylaxis was based on cyclosporine and short term methotrexate. The graft cell dose was: TNC 5.82 × 10^8^/kg and CD34+ 2.62 × 10^6^/kg. On day +5 the child presented sub-acute onset of headache, lethargy, confusion, slurred speech, and left-hemibody hyposthenia. The electroencephalogram (EEG) showed a continuous ictal epileptic discharge involving the right hemisphere (Figure 2A), consistent with the diagnosis of focal status epilepticus (SE), successfully treated with intravenous administration of levetiracetam and phenytoin. Once SE was controlled, a residual confusional state was observed with EEG, showing an encephalopathic pattern characterized by diffuse slowing clearly predominant on the right hemisphere and periodic temporo-occipital sharp transients (Figure 2B). Brain MRI showed multiple focal hyperintense lesions in T2-weighted (Figures 2E–H) and FLAIR (Figures 2I–P) sequences, localized in corpus callosum (Figures 2E–P), right posterior thalamus (Figures 2I,M) and at the cortico-subcortical junction bilaterally(Figures 2J,O), mainly on the right hemisphere. The cerebrospinal fluid (CSF) findings showed elevated protein concentration while it resulted negative for bacterial and viral infections. Serum and CSF analysis resulted negative for anti-glutamic acid decarboxylase antibodies (anti-GAD), anti-NMDAR, anti-AMPA1, anti-AMPA2, anti-CASPR2, anti-LGI1, anti-GABARB1/B2, anti-myelin oligodendrocyte glycoprotein antibodies (anti-MOG), while the presence of oligoclonal bands was detect only in CSF. The patient was treated with intravenous immunoglobulins (2 g/kg) with gradual clinical improvement in 3 days and complete resolution of symptoms within a week. The EEG performed after 10 days from onset demonstrated a slight inter-hemispheric asymmetry, due to excessive slow activities on the right posterior regions (Figure 2C). A follow-up MRI performed 30 days after the encephalitic episode showed a complete resolution of the focal lesions (Figures 2Q–T). After a primary myeloid engraftment on day +13 (peak neutrophil count of 5.400/mm^3^), the patient had another early secondary graft failure on day +30 (0% donor-derived cells). Despite that, since day +30 the girl had a progressive recovery of blood values and she no longer needed transfusions, maintaining a normal blood count on erythropoietin treatment. A bone marrow aspirate performed 100 days after the second transplant showed a normal trilineage haematopoiesis with the presence of erythroid precursors (Figure 3), whereas the chimerism confirmed a complete recipient reconstitution (0% donor-derived cells). The EEG performed 3 months after encephalitis showed a complete normalization of previous pathological findings (Figure 2D). At her last follow-up visit on day +150 after the second transplant, the child is in excellent general condition (Lansky play score 100%) with a normal blood count and transfusion-independency although supported with erythropoietin (10,000 UI once every 2 weeks).

## Discussion

APS-1 is characterized by a marked clinical variability. The risk to develop organ-specific autoimmune manifestations depends upon both environmental factors and genetic susceptibility (2). We described two sisters with the same AIRE mutation but different phenotypes. The older girl (case 1) presented a classic pattern of the disease, including the three cardinal characteristics: chronic mucocutaneous candidiasis, hypoparathyroidism, and adrenal insufficiency. The younger girl presented some atypical features of the disease like PCRA and cerebellar hypoplasia. PRCA is characterized by normocytic anemia, reticulocytopenia, absence of erythroid precursors in the bone marrow with normal values of white blood cells and platelets. It can be considered as the result of antibody-mediated or T-cell-mediated damage of the erythroid progenitor cell with an inhibition of erythropoiesis (9). The role of γδ T-cell receptor positive lymphocytes in the pathogenesis of the PRCA associated with APS-1 is described in several papers (9, 10). Only 8 cases of PRCA associated with APS-1 have been reported in the literature. In most cases the onset of PRCA occurs during the course of the disease, in adulthood, after the development of the typical manifestations of the APS-1 (8–12). In literature only 3 children with PRCA and APS-1 have been reported. In one of the cases [a 10 year-old girl (13)] PRCA developed after the onset of the typical characteristics of APS-1; in the other described cases (a 7 year-old and a 15 year-old patient) PRCA was the prominent feature of the APS-1 (14). In our case as well PRCA was the main manifestation of the disease and began before adrenal insufficiency. The diagnosis of PRCA was made at the age of 3: this is the earliest onset age reported in the literature. The therapy of PRCA associated with APS-1 is currently not standardized due to the rarity of the disease. In the cases reported in the literature the therapy that was used with good results was corticosteroid (12–14), cyclophosphamide (11–13), mycophenolate mofetil (10), plasmapheresis (13), immunoglobulin (8, 13, 14), cyclosporine (8), antithymocyte globulin (ATG) (8). In our patient, no benefit was obtained by treatment with immunoglobulin, corticosteroid, and erythropoietin therapy. The child became transfusion-dependent and was referred to receive an HSCT. She represents the first case of PRCA associated with APS-1 undergoing HSCT. In literature there are no reported cases of APS-1 with cerebellar hypoplasia, but it is described in association with hereditary bone marrow failure like Fanconi anemia (15), dyskeratosis congenital (16), Diamond-Blackfan anemia (17), congenital amegakaryocytic thrombocytopenia (18), or ataxia-pancytopenia syndrome (19). Some cases of patients with cerebellitis and APS-1 are described (20); in these patients high serum and CSF titers of anti-GAD were found, with intrathecal production of such antibodies. In our patient serum and CSF analysis were performed after the transplantation, when the child developed an encephalitis, and they resulted negative for all autoantibodies against neuronal surface and intracellular antigens but positive for oligoclonal bands in CSF. This finding probably suggests an immune-mediated pathogenesis of the damage which developed after HSCT, as described in some other papers (21–23). Neurologic complications after HSCT are relatively common, but demyelinating disease is rare (23). The features of MRI lesions are highly suggestive of an acute disseminated encephalomyelitis (ADEM). In our case the onset of neurological symptoms is very close to the II HSCT so we suppose that the damage is a consequence of the I HSCT. In literature, the reported mean time from HSCT to the beginning of symptoms of ADEM is about 120 days (23); in our case the symptoms appeared 47 days after the first HSCT and 5 days after the second one. Therapy with immunoglobulin was shown to be effective with prompt clinical response and disappearance of MRI lesions, as already reported (21–24). The child is currently in clinical and hematological remission of disease, transfusion-indipendent with normal blood count despite graft failure occurred twice as documented by the complete patient myeloid recovery at chimerism analysis. Given the fact that hematological remission have been described in patients with PRCA associated with APS-1 treated with cyclophosphamide (11, 12), we hypothesize that the recovery of erithropoiesis observed after the second HSCT was the immunosuppressive effect of cyclophosphamide, included in the conditioning regimen. In conclusion, we found out that APS-1 has a significant clinical heterogeneity despite the same genetic mutation and this has to be considered in the differential diagnosis of pure red cell aplasia. In our experience, this condition might be treated with a therapeutic regimen that includes the use of the ATG and the cyclophosphamide as severe aplastic anemia, while the HSCT is characterized by a high risk of graft failure. Finally, our case showed the efficacy of immunoglobulin therapy in a case of ADEM post HSCT.

## Data Availability

The datasets generated for this study can be found in U.O servizio di anatomia patologica, Università degli Studi di Brescia, GM2018-000457.

## Ethics Statement

Written informed consent was obtained from patients' parents for publication of this case report and any potentially-identifying information/images. The work was exempt from ethics committee review/approval.

## Author Contributions

MC involvement in medical diagnosis and follow up of patients, first writer of the manuscript. MM and GC involvement in medical diagnosis of patients, they helped to write the manuscript. RB, MD, VV, AZ, EB, RG, and EF involvement in diagnosis and management of patients. SC involvement in diagnosis and management of patients, supervision of the process of the manuscript. All authors read and approved the final manuscript.

### Conflict of Interest Statement

The authors declare that the research was conducted in the absence of any commercial or financial relationships that could be construed as a potential conflict of interest.
